# Multifocal and Multicentric Glioblastoma with Leptomeningeal Gliomatosis: A Case Report and Review of the Literature

**DOI:** 10.1155/2013/132679

**Published:** 2013-12-05

**Authors:** Sophia F. Shakur, Esther Bit-Ivan, William G. Watkin, Ryan T. Merrell, Hamad I. Farhat

**Affiliations:** ^1^Section of Neurosurgery, University of Chicago Medical Center, Chicago, IL 60637, USA; ^2^Department of Pathology, NorthShore University HealthSystem, Evanston, IL 60201, USA; ^3^Department of Neurology, NorthShore University HealthSystem, Evanston, IL 60201, USA; ^4^Department of Neurosurgery, NorthShore University HealthSystem, 2650 Ridge Avenue, Evanston, IL 60201, USA

## Abstract

Glioblastoma (GBM) rarely presents as an infratentorial tumor in adults. The authors present a case of concomitant supratentorial and infratentorial GBM in an adult. A 72-year-old man presented with headache, nausea, vomiting, and lightheadedness. Initial MR images revealed enhancing masses in the right cerebellum and right posterior periventricular region. The patient underwent a suboccipital craniotomy and resection of the cerebellar lesion. Final histopathology was consistent with glioblastoma. The patient went on to receive standard radiation treatment for GBM with concurrent and adjuvant temozolomide. However, the patient experienced clinical deterioration within a few days after starting radiotherapy. He and his family decided to forego treatment and pursue palliative care. The patient expired three months after the initial diagnosis. Autopsy findings supported the diagnosis of GBM with leptomeningeal gliomatosis and involvement of the cerebrum, cerebellum, and spinal cord. The authors review the literature and propose that the pathogenesis of multiple and multicentric GBM may involve neural stem cells within the subventricular zone or could result from tumor dissemination along established CNS routes, such as white matter tracts and CSF pathways.

## 1. Introduction

Glioblastoma (GBM) is the most common intraparenchymal primary brain tumor, representing approximately 30% of all brain tumors and 50% of astrocytomas [1, 2]. GBM is usually located in the deep white matter of the frontal and temporal lobes and rarely occurs in the brainstem, cerebellum, and spinal cord [3]. Despite surgical resection and intensive adjuvant radiotherapy and chemotherapy, the median survival following the diagnosis of GBM is only 14.6 months [4]. In 90% of cases, tumor recurrence is found within a margin of 2 to 3 cm from the original tumor site [5, 6]. Our understanding of the biological behavior of GBM remains limited.

Multiple gliomas were first observed by Virchow in 1864 and Bradley in 1880 [7–9]. In their seminal paper published in 1962, Batzdorf and Malamud characterized the modes of growth in gliomas by establishing criteria to distinguish multiple and multicentric gliomas [10]. Namely, multiple glioma disseminates along established CNS routes, such as white matter tracts, cerebrospinal fluid (CSF), or local invasion. In contrast, multicentric glioma is widely separated in location and/or time. The incidence of solitary, multiple, and multicentric gliomas in their series of 209 gliomas was 72.2%, 25.4%, and 2.4%, respectively. Since then, others have attempted additional classifications based on pathologic and radiologic criteria [8, 11]. Multifocal replaces multiple in the modern day classification. Therefore, multifocal glioma consists of tumors separated by white matter tracts within the same hemisphere, whereas multicentric glioma consists of tumors in opposite hemispheres or separated by the tentorium. Multicentric GBM involving the supratentorial and infratentorial regions is even more rare [8, 12].

Here, we report a case of a multifocal and multicentric GBM involving the supratentorial and infratentorial regions in an adult and review the literature on all previously documented cases. We also discuss the pathogenesis of this unique presentation of GBM, thereby providing greater insight into the origin and growth of GBM. This insight may lead to changes to the treatment approach of this rare presentation of GBM.

## 2. Case Report

### 2.1. History and Examination

**Figure 1:**
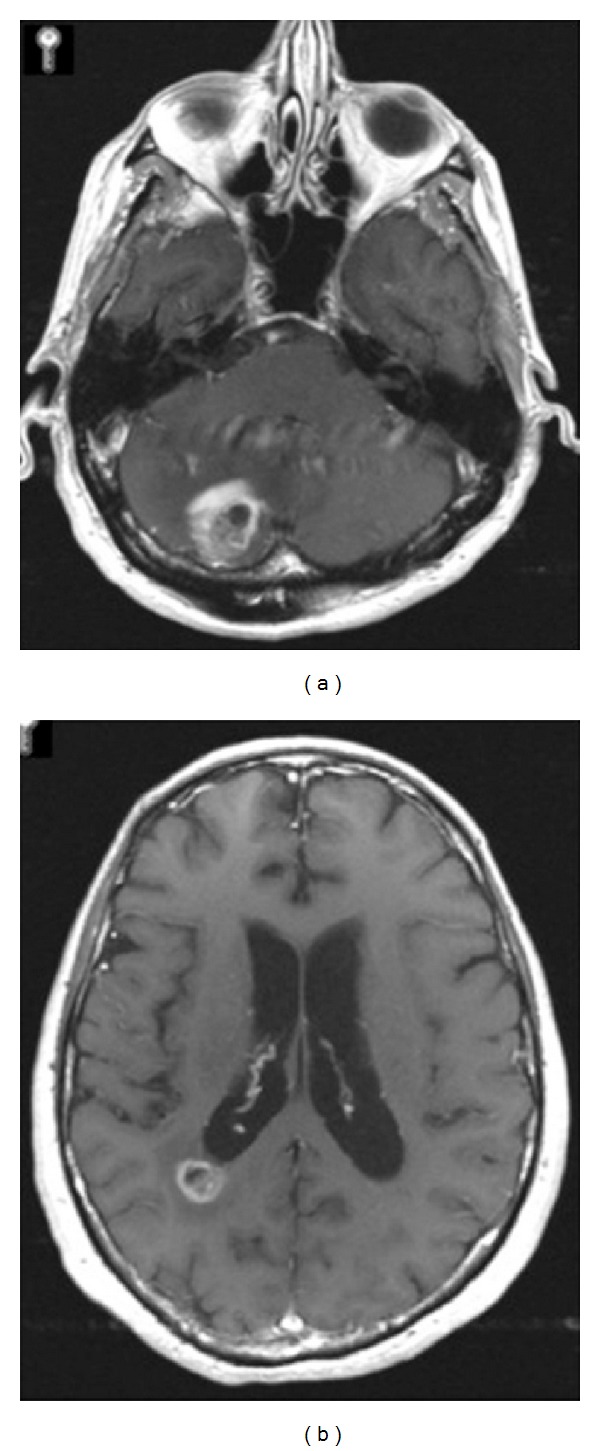
Preoperative MR imaging. (a) Axial T1-weighted image with contrast at the level of the cerebellum showing an enhancing mass in the right cerebellar hemisphere measuring 2.9 cm × 2.4 cm. (b) Axial T1-weighted image with contrast at the level of the lateral ventricles demonstrating an enhancing lesion in the right posterior periventricular region measuring 1.0 cm × 1.4 cm.

A 72-year-old right-handed male presented to the Emergency Department with headache, nausea, vomiting, and lightheadedness of approximately one week duration. On neurological examination, the patient was intact without any focal deficits. Computed tomography (CT) of the head showed multifocal areas of cerebral edema with areas of abnormal increased attenuation, predominantly involving the right cerebellar hemisphere and adjacent to the right lateral ventricle posteriorly. The cerebellar edema was associated with mass effect on the fourth ventricle and obstructive hydrocephalus. These findings were most suspicious of brain metastases. Subsequent magnetic resonance (MR) imaging of the brain with and without contrast revealed enhancing masses in the right cerebellum measuring 2.9 cm × 2.4 cm and in the right posterior periventricular region measuring 1.0 cm × 1.4 cm as well as subtle areas of abnormal enhancement adjacent to the temporal horn of the right lateral ventricle (Figure 1). The patient was admitted to the intensive care unit (ICU), started on dexamethasone, and scheduled for surgical resection of the right cerebellar mass. Body CT demonstrated no apparent primary neoplasm.

### 2.2. Operation

**Figure 2:**
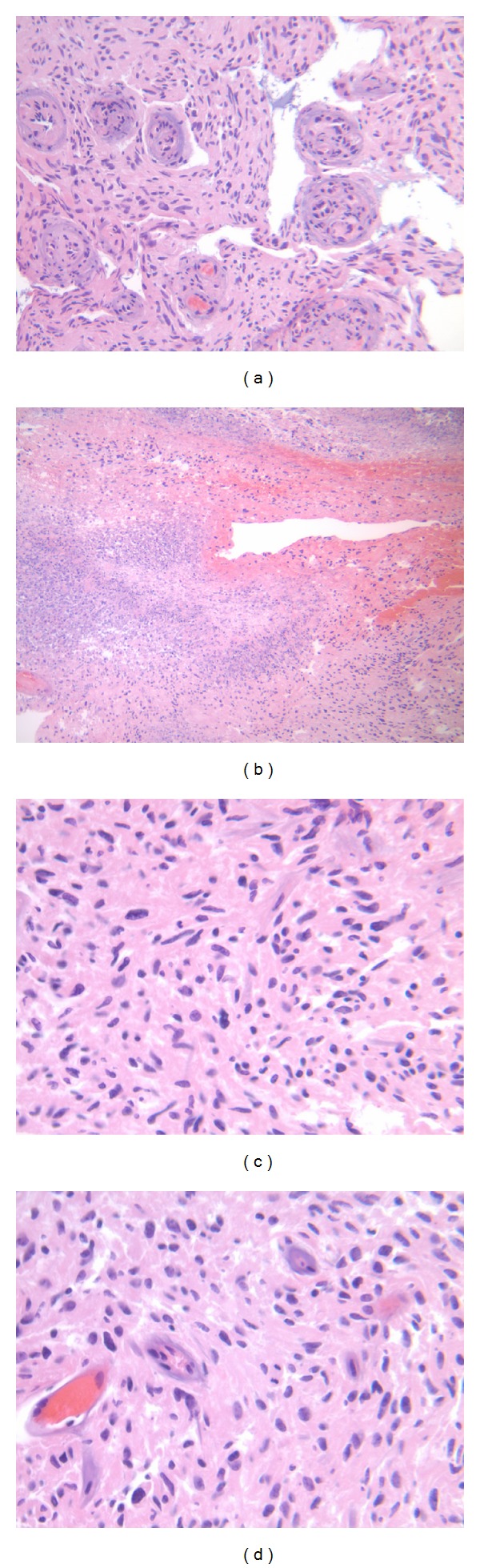
Photomicrographs displaying pathognomonic histopathological features of glioblastoma. (a) Microvascular proliferation with endothelial layer hyperplasia. (b) Areas of geographic necrosis are present. (c) Microscopic examination reveals a hypercellular neoplasm with large, hyperchromatic, pleomorphic cells with cerebellar granular layer cells on the left. (d) Mitotic figures are readily identified in a background of pleomorphic, hyperchromatic neoplastic cells. H&E; original magnification ×10 (a), ×10 (b), ×10 (c), and ×40 (d).

**Figure 3:**
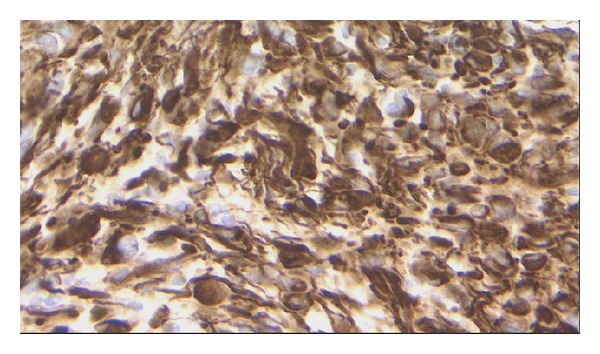
Photomicrograph demonstrating results of GFAP immunohistochemical staining. The neoplastic cells are strongly positive for GFAP immunostain. Original magnification ×40.

The patient underwent a suboccipital craniotomy and resection of the cerebellar lesion. Final histopathology was consistent with glioblastoma (Figure 2). Specifically, histologic sections demonstrated a hypercellular proliferation of pleomorphic, hyperchromatic astrocytes accompanied by microvascular proliferation and palisading necrosis. Numerous mitotic figures were present. Glial fibrillary acidic protein (GFAP) was strongly positive in the tumor cells (Figure 3).

### 2.3. Postoperative Course

**Figure 4:**
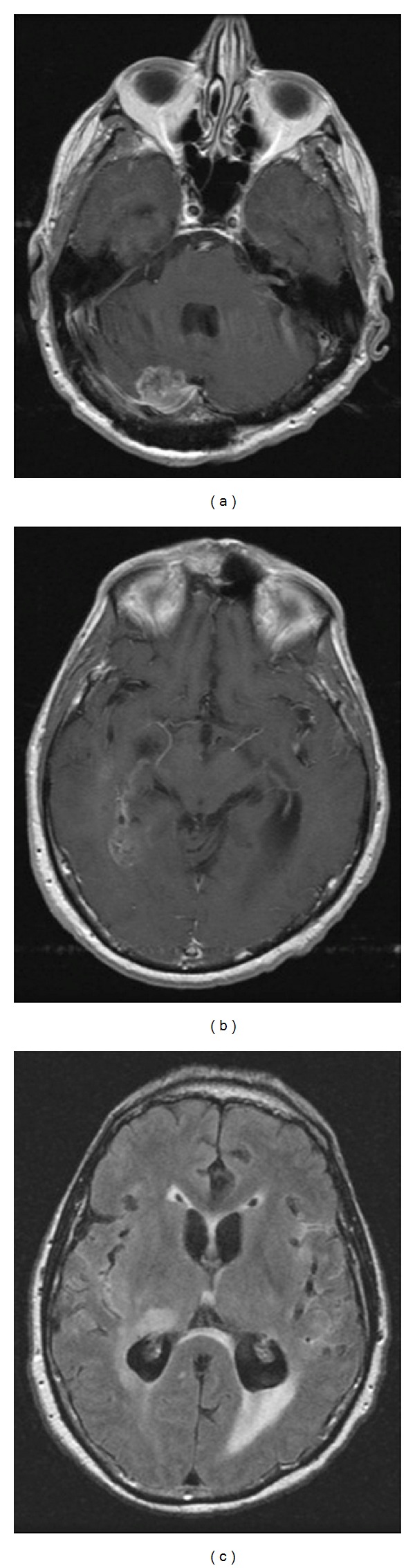
Postoperative MR imaging. (a) Axial T1-weighted image with contrast at the level of the cerebellum displaying subtotal resection of the tumor within the right cerebellar hemisphere. (b) Axial T1-weighted image with contrast at the level of the Sylvian fissures showing leptomeningeal enhancement. (c) Axial FLAIR image above the tentorium revealing diffuse FLAIR signal abnormality within bilateral cerebral hemispheres.

Postoperative MR imaging showed leptomeningeal enhancement within the Sylvian fissures and diffuse FLAIR signal abnormality within bilateral cerebral hemispheres (Figure 4). The patient went on to receive fractionated intensity modulated radiation therapy (IMRT) encompassing a wide field to treat the enhancing as well as the nonenhancing areas of tumor along with concomitant daily temozolomide (75 mg/m^2^). The patient experienced clinical deterioration with headaches and mental status changes within a few days of starting radiotherapy. He and his family decided to forego treatment and pursue palliative care. The patient expired at home two months later.

### 2.4. Autopsy

**Figure 5:**
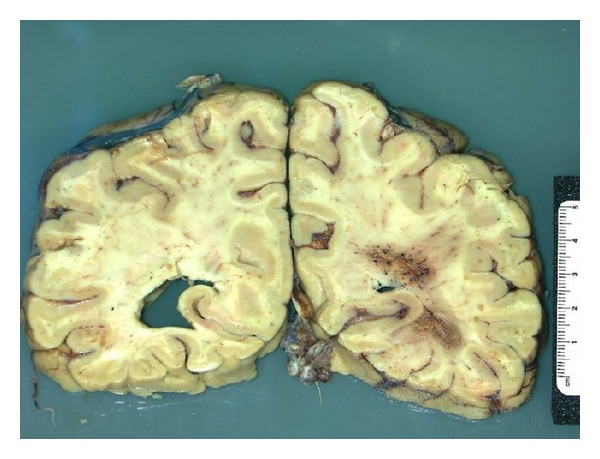
Gross autopsy image. A coronal section of the brain showing two grossly distinct, hemorrhagic lesions adjacent to the occipital horn of the right lateral ventricle associated with ventricular compression.

Autopsy findings supported the diagnosis of GBM with leptomeningeal gliomatosis and involvement of the cerebrum, cerebellum, spinal cord, neurohypophysis, and choroid plexus. In particular, two grossly separate lesions were present within the right hemisphere of the cerebrum, adjacent to the occipital horn of the right lateral ventricle, which were microscopically connected by infiltrating tumor cells and were consistent with GBM (Figure 5). Tumor cells were also found in the leptomeninges of bilateral Sylvian fissures and spinal cord as well as parts of the choroid plexus and neurohypophysis. The original cerebellar resection specimen, the cerebral tumor, and leptomeningeal lesions were stained with *p53 *and were all focally positive.

## 3. Discussion

In this paper, we report a case of multifocal and multicentric supratentorial and infratentorial GBM in an adult. Our review of the pertinent literature revealed that only 11 other cases of concomitant supratentorial and infratentorial GBM have been documented. The clinical findings in all published adult cases of supratentorial and infratentorial GBM are summarized in Table 1. The age at diagnosis ranged from 24 to 74 years (median 51.5 years). Ten patients were male and two were female. Infratentorial tumors occurred in the cerebellar hemisphere (5 cases), vermis (3 cases), brainstem (3 cases), and cerebellopontine angle (1 case). Histopathological analysis of the supratentorial and infratentorial masses matched in 9 cases and was mixed in 3 cases. In this series, spanning 5 decades with varying management strategies, overall survival ranged from 2 to 18 months (median 4 months).

Previously, multifocal and multicentric GBM have been associated with a worse prognosis than solitary GBM, with median patient survival estimates of 6–8 months after different treatment modalities [13–15]. A recent study found a statistically significant difference in median survival between patients with multifocal GBM and solitary GBM of 9.6 months versus 14.6 months (*P* = 0.014) but not between patients with multicentric GBM and solitary GBM (12.9 months and 14.6 months, resp.) [15]. Tumor dissemination at the time of diagnosis can also serve as a prognostic marker. One study stratified patients into three groups based on solitary or multifocal tumors and whether there was subependymal or subarachnoid dissemination at the time of diagnosis [16]. Patients with multifocal tumors and subependymal and subarachnoid dissemination had shorter progression free and overall survival than patients with solitary tumors with subependymal and subarachnoid dissemination. However, patients with multifocal tumors without subependymal and subarachnoid dissemination had similar outcomes to patients with solitary GBM.

The radiographic appearance of multifocal and multicentric GBM is indistinct from that of metastases, with MR imaging displaying multiple contrast-enhancing masses [3, 12, 17]. Certain MR imaging features such as variable lesion morphology, mild peritumoral edema, and irregular tumor margins can suggest the diagnosis of multiple or multicentric GBM [15, 18, 19]. Since metastasis from extracranial primary tumors is the most common diagnosis associated with multiple brain masses and the most common intra-axial posterior fossa tumor in adults, histopathological verification is imperative before making a diagnosis of metastasis, especially in patients with no known primary neoplasm [3, 17].

The diagnostic workup for multifocal and multicentric GBM is generally the same as that for solitary GBM. Dissemination of GBM, however, can occur intracranially or throughout the spinal axis [16]. In such cases of GBM with leptomeningeal gliomatosis, either suspected clinically or radiographically due to the presence of leptomeningeal enhancement or hydrocephalus, complete neuroaxis MR imaging should be obtained. Cytological examination of the CSF can be used to confirm the diagnosis. Additional treatment modalities available to these patients are outlined later in this discussion.

Multifocal and multicentric GBM do not exhibit any histopathologic characteristics that differentiate them from typical, solitary supratentorial GBM [10, 20]. Pathognomonic features of GBM include pseudopalisading necrosis and neovascularization [21]. The individual tumors in cases of multicentric GBM usually have the same pathologic appearance [10, 22].

Although there is still no unified theory regarding the pathogenesis of multifocal and multicentric GBM, several hypotheses have been developed. According to earlier theories, multicentricity arises from two events [8, 10]. The first stage is neoplastic transformation, in which a wide field becomes more susceptible to neoplastic growth. The second stage is tumor proliferation at two or more activated sites that can occur simultaneously in response to various stimuli including biochemical, hormonal, and viral triggers. More contemporary theories have looked at molecular associations. For example, there is a reported association between *p53* mutations and multifocal GBM that correlates the pattern of *p53* mutation to tumor migration and augmented growth [23, 24]. In a study of the growth factor receptor c-Met in GBM, one group found that 42.9% of tumors that overexpressed c-Met displayed invasive and multifocal features on initial MR imaging, whereas only 17.1% of tumors with little or no c-Met expression had similar characteristics (*P* = 0.036) [25]. This molecular and genetic characterization of multifocal GBM has thereby implicated particular oncogenes and growth factors in the pathogenesis of multifocal and multicentric GBM.

In addition to the molecular pathways involved in multifocality and multicentricity, studies have correlated the tumor pattern at diagnosis and recurrence with the spatial relationship to the subventricular zone (SVZ) and cortex as seen on MR imaging [26]. More specifically, patients with a contrast-enhancing lesion contacting the SVZ and infiltrating the cortex were most likely to have multifocal disease at the time of diagnosis and distant tumor recurrence. On the other hand, patients with a contrast-enhancing lesion neither contacting the SVZ nor infiltrating the cortex always had solitary lesions and contiguous tumor recurrences. Neural stem cells within the SVZ may give rise to multiple and multicentric GBM. Neural stem cells have been found to express matrix metalloproteinases, which are proteolytic enzymes implicated in tumor spread [26]. Furthermore, the SVZ is thought to be a highly permissive environment for tumor growth and cellular migration.

Currently, there are no clear guidelines regarding the optimal management of multifocal and multicentric GBM [15]. While the extent of resection is established as an independent determinant of survival in patients with solitary GBM, the role of surgery for multifocal and multicentric GBM remains controversial [6, 15, 27]. Aggressive resection of one tumor focus, biopsy alone followed by chemotherapy and radiation treatment, and multiple craniotomies during a single operation have all been described with no clear indication of which modality is superior [15, 28–30]. The standard radiation treatment for GBM includes conformal radiotherapy that encompasses the tumor volume and margin along with concurrent and adjuvant temozolomide [4, 30, 31]. Regarding radiotherapy for multifocal and multicentric GBM, a study found no significant difference in the median time to progression or median survival time between conformal radiotherapy and whole brain radiation treatment [30]. Additionally, the role of radiotherapy to control infratentorial GBM is not yet defined [32–34]. Some studies support the use of craniospinal radiotherapy with posterior fossa boost for malignant cerebellar gliomas, especially in children [33]. However, other studies have found no benefit with craniospinal radiotherapy in GBM in adults and support whole brain radiation treatment with or without posterior fossa boost [32]. Most authors agree that craniospinal irradiation is reasonable if CSF dissemination occurs [34, 35]. In patients with leptomeningeal gliomatosis, other treatments could also include intrathecal chemotherapy and CSF shunting for associated hydrocephalus.

## 4. Conclusions

In our case, we describe an adult with concomitant supratentorial and infratentorial GBM. The patient expired three months after the initial diagnosis. Autopsy findings supported the diagnosis of GBM with leptomeningeal gliomatosis. Only 11 such cases have been previously published in the literature. Tumor dissemination could have occurred through seeding of the CSF by the periventricular mass. Alternatively, the supratentorial tumor location at the SVZ could have served as a nidus for dissemination along CNS pathways. Molecular characterization of the tumor with immunohistochemical staining for *p53* was positive in both the cerebellar and cerebral lesions. Expression of c-Met was not determined.

Currently, there is no specific treatment protocol for multiple or multicentric GBM. Unfortunately, these patients are often excluded from clinical trials. Our patient had a rapid clinical decline despite standard treatment and elected to forego additional treatment. Whether he may have benefitted from an alternative treatment approach with targeted molecular agents is uncertain.

Our compilation and assessment of the case reports on supratentorial and infratentorial GBM in adults documented to date yield several conclusions. This presentation of GBM is rare. MR imaging characteristics are similar to metastatic tumors and therefore histopathological confirmation is necessary. Concomitant supratentorial and infratentorial GBM has the same histological appearance as that of solitary GBM. Finally, molecular and genetic analysis of these unique tumors may provide insight into their pathogenesis as well as the origin and growth of GBM in general.

## Figures and Tables

**Table 1:** Literature review of studies on concomitant supratentorial and infratentorial glioblastomas in adults*.

Authors and year	Case no.	Age (yrs), sex	Location		Histopathology		Treatment	Overall survival (mos)
			Supratentorial	Infratentorial	Supratentorial	Infratentorial		
Present study	1	72, M	Rt posterior periventricular region	Rt cerebellar hemisphere	Glioblastoma	Glioblastoma	STR, TMZ, and WBXRT	3

Salunke et al. [34], 2010	2	50, M	Rt insula	Cerebellar vermis	Grade II astrocytoma	Glioblastoma	STR, WBXRT	18

Kotwica and Papierz, 1992 [36]	3	53, F	Lt temporal lobe	Cerebellar vermis	Glioblastoma	Pilocytic astrocytoma	GTR	10

Salvati et al. [8], 1991	4	47, M	Rt frontal lobe	Lt cerebellar hemisphere	Glioblastoma	Grade III astrocytoma	Biopsy	2
	5	42, M	Rt temporal lobe	Pons	Glioblastoma	Glioblastoma	GTR	Postoperative mortality
	6	56, F	Lt frontoparietal lobes	Rt cerebellar hemisphere	Glioblastoma	Glioblastoma	Biopsy, Chemotx, and WBXRT	7

Kudo et al. [12], 1990	7	74, M	Rt occipital lobe	Rt cerebellar hemisphere	Glioblastoma	Glioblastoma	STR	2

Bussone et al. [20], 1979	8	49, M	Rt thalamus	Rt CPA	Glioblastoma	Glioblastoma	None	4

Takeda et al. [9], 1976	9	56, M	Corpus callosum	Cerebellar vermis	Glioblastoma	Glioblastoma	Unknown	3

Ishida and Nakagawa [37], 1972	10	53, M	Rt frontal lobe	Brainstem	Glioblastoma	Glioblastoma	Unknown	18

Salles et al. [38], 1967	11	24, M	Lt temporal lobe	Lt cerebellar hemisphere	Glioblastoma	Glioblastoma	Unknown	8

Kijima et al. [39], 1962	12	31, M	Rt temporal lobe	Brainstem	Glioblastoma	Glioblastoma	Unknown	3

*STR: subtotal resection; GTR: gross-total resection; TMZ: temozolomide; WBXRT: whole brain radiotherapy; Chemotx: chemotherapy; CPA: cerebellopontine angle.
